# Supplementary material for: Modeling glioblastoma heterogeneity as a dynamic network of cell states
Source: Mol Syst Biol. 2021 Sep 16;17(9):e10105. doi: 10.15252/msb.202010105 (PMC8444284; doi:10.15252/msb.202010105)
Supplement: Supplementary file 6 — Source Data for Figure 5 [file MSB-17-e10105-s004.zip › Figure5A_sourcedata/GSEA_3071/hallmarks_stateVI.GseaPreranked.1623742557031/HALLMARK_CHOLESTEROL_HOMEOSTASIS.html]

Details for gene set HALLMARK\_CHOLESTEROL\_HOMEOSTASIS[GSEA]

|  || Dataset | state63071 |
| Phenotype | NoPhenotypeAvailable |
| Upregulated in class | na\_neg |
| GeneSet | HALLMARK\_CHOLESTEROL\_HOMEOSTASIS |
| Enrichment Score (ES) | -0.26966232 |
| Normalized Enrichment Score (NES) | -0.95202386 |
| Nominal p-value | 0.5135135 |
| FDR q-value | 0.71837574 |
| FWER p-Value | 1.0 |
Table: GSEA Results Summary

  

Fig 1: Enrichment plot: HALLMARK\_CHOLESTEROL\_HOMEOSTASIS      
 Profile of the Running ES Score & Positions of GeneSet Members on the Rank Ordered List

  

| PROBE | GENE SYMBOL | GENE\_TITLE | RANK IN GENE LIST | RANK METRIC SCORE | RUNNING ES | CORE ENRICHMENT || 1 | ALCAM |  |  | 7 | 1.185 | 0.0926 | No |
| 2 | PLAUR |  |  | 34 | 0.936 | 0.1601 | No |
| 3 | TRIB3 |  |  | 387 | 0.495 | 0.1023 | No |
| 4 | ATF5 |  |  | 401 | 0.490 | 0.1378 | No |
| 5 | ATF3 |  |  | 416 | 0.483 | 0.1725 | No |
| 6 | ACTG1 |  |  | 508 | 0.453 | 0.1835 | No |
| 7 | ERRFI1 |  |  | 526 | 0.449 | 0.2146 | No |
| 8 | JAG1 |  |  | 876 | 0.365 | 0.1472 | No |
| 9 | NFIL3 |  |  | 956 | 0.352 | 0.1535 | No |
| 10 | GPX8 |  |  | 1112 | 0.328 | 0.1369 | No |
| 11 | ANTXR2 |  |  | 1183 | 0.318 | 0.1429 | No |
| 12 | FDFT1 |  |  | 1552 | 0.274 | 0.0630 | No |
| 13 | CHKA |  |  | 1733 | 0.257 | 0.0338 | No |
| 14 | SREBF2 |  |  | 2032 | -0.283 | -0.0260 | No |
| 15 | CTNNB1 |  |  | 2863 | -0.483 | -0.2169 | No |
| 16 | PNRC1 |  |  | 3055 | -0.566 | -0.2245 | Yes |
| 17 | FABP5 |  |  | 3079 | -0.577 | -0.1848 | Yes |
| 18 | FADS2 |  |  | 3173 | -0.640 | -0.1595 | Yes |
| 19 | TM7SF2 |  |  | 3334 | -0.802 | -0.1397 | Yes |
| 20 | CD9 |  |  | 3445 | -0.995 | -0.0907 | Yes |
| 21 | CLU |  |  | 3601 | -1.801 | 0.0102 | Yes |
Table: GSEA details [plain text format]

  

Fig 2: HALLMARK\_CHOLESTEROL\_HOMEOSTASIS: Random ES distribution      
 Gene set null distribution of ES for **HALLMARK\_CHOLESTEROL\_HOMEOSTASIS**
